# From Waste to Green Applications: The Use of Recovered Gold and Palladium in Catalysis

**DOI:** 10.3390/molecules26175217

**Published:** 2021-08-28

**Authors:** Sean McCarthy, Alvin Lee Wei Jie, D. Christopher Braddock, Angela Serpe, James D. E. T. Wilton-Ely

**Affiliations:** 1 Department of Chemistry, Imperial College London, Molecular Sciences Research Hub, White City Campus, London W12 0BZ, UK; s.mccarthy19@imperial.ac.uk; 2 Department of Civil and Environmental Engineering and Architecture, INSTM Unit, University of Cagliari, Via Marengo 2, 09123 Cagliari, Italy; alvinl.w.j95@gmail.com

**Keywords:** recycling, gold, palladium, catalysis, WEEE, TWC, critical metals, circular economy, green processes

## Abstract

The direct use in catalysis of precious metal recovery products from industrial and consumer waste is a very promising recent area of investigation. It represents a more sustainable, environmentally benign, and profitable way of managing the low abundance of precious metals, as well as encouraging new ways of exploiting their catalytic properties. This review demonstrates the feasibility and sustainability of this innovative approach, inspired by circular economy models, and aims to stimulate further research and industrial processes based on the valorisation of secondary resources of these raw materials. The overview of the use of recovered gold and palladium in catalytic processes will be complemented by critical appraisal of the recovery and reuse approaches that have been proposed.

## 1. Introduction

Gold and palladium are precious metals that occupy an important place in numerous industries. Indeed, besides their historical applications in jewellery and as an investment, they play a key role in many new cutting-edge technologies. Owing to its excellent conductivity and stability, gold is extensively applied in electronics; palladium is well known for its potent catalytic properties in a variety of organic reactions and is exploited as a stable oxidation catalyst in three-way catalytic converters (TWCs). Unrelenting demand for these metals has resulted in record prices and their supply is plagued with concerns over the environmental impact of their production and finite natural supply [1,2,3,4,5]. Consequently, over the last 20 years, manufacturers and researchers have been focusing their efforts on limiting or avoiding the use of precious metals in specific applications and/or seeking more sustainable sources of these metals.

One of the fields that is susceptible to the ‘critical’ nature of gold and palladium is the catalytic transformation of organic substrates, which underpins the synthesis of pharmaceuticals and fine chemicals. There has been an intense effort in the last decade to replace these metals with less expensive and more abundant metals such as cobalt, nickel, iron, and copper, among others [6,7,8]. Unfortunately, the performance of such catalysts is often unsatisfactory both in terms of catalytic activity and stability, making them less appealing for industrial purposes [9,10,11]. For this reason, the possibility of using precious metal compounds derived from waste processing directly in catalysis has experienced a growing interest, particularly in the academic community. This has led to greater focus on waste electric and electronic equipment (WEEE) and end-of-life vehicles, which represent the richest secondary sources of gold and palladium, respectively. This concept of ‘urban mining’ provides a potential solution to waste disposal issues, the dwindling sources of the metals and the environmentally damaging mining practices currently used to extract them.

This review provides an overview of the published research on the use of recovered gold and palladium compounds in catalysis in the context of the industrially established recovery processes and products. Innovative, earlier-stage yet promising approaches to improve the sustainability of the circular economy model will also be covered to provide suggestions for future research in the area.

## 2. Gold

### 2.1. Primary Production of Gold

The global supply of gold is predominantly sourced from mining, typically amounting to 60–75% of supply each year. Since annual demand for gold exceeds mining capacity, the shortfall is made up from gold recycling [12]. Hard rock mining is responsible for most of the gold produced in the world today and this is mainly carried out underground [13]. Underground mining is by far the most arduous and dangerous technique, as vertical tunnels must be dug and shafts drilled to access the lode [13,14,15]. Other than hard rock mining, gold is also produced from open-pit mining and by-product mining, where gold is found alongside other material [13,16].

After being extracted from the ground, unrefined ores must be furtherly processed to fully recover the gold deposit. Gold cyanidation is currently the main method used to process gold ores. This technique employs basic leaching solutions of sodium or potassium cyanide in the presence of oxygen to extract precious metals from the ore [17], according to the Elsner Equation (Equation (1)).

4 Au _(s)_ + 8 CN^−^ _(aq)_ + O_2 (g)_ + 2 H_2_O _(l)_ → 4[Au(CN)_2_]^−^ _(aq)_ + 4 OH^−^ _(aq)_ (1)

The gold leaching product is then typically isolated by adsorption on activated carbon followed by stripping steps, before gold metal is deposited by electrowinning or cementation. As an alternative, cementation with zinc may be directly applied to the leachate. In both cases, due to the complexity of the ore composition and the limited selectivity of the leaching process, the recovered solid materials undergo smelting and separation for refining [17,18]. Despite its effectiveness, the cyanidation process requires strict control over the pH to prevent the formation of deadly gaseous HCN. The process generates a huge volume of waste called ‘tailings’, which may contain cyanide and a range of toxic heavy metals, such as mercury, cadmium, and lead [19,20]. This has led to many governments and the EU imposing mining waste directives and limits, such as for the concentration of cyanide in waste streams (typically these must be lower than 10 ppm). In some countries, such as Germany, Hungary, and Turkey, gold cyanidation is completely banned due to its adverse environmental impacts.

**Figure 1:**
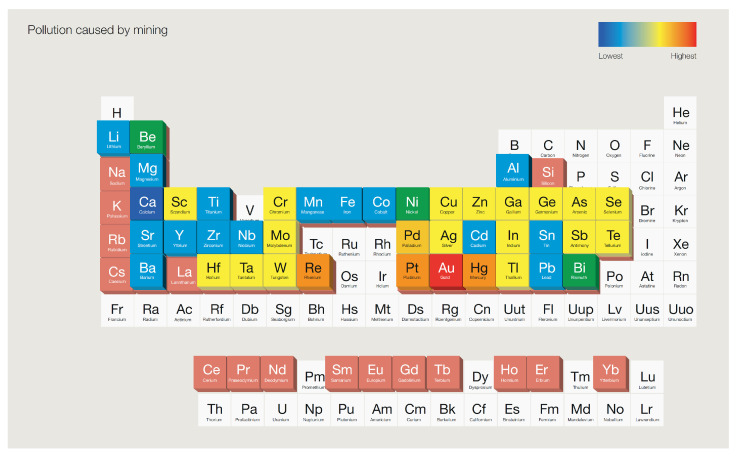
Periodic table depicting the pollution impact caused by mining of various elements. Reproduced with permission from [22].

For similar reasons, amalgamation, an historic process using mercury to recover and concentrate gold (or silver) from ores, was phased out from large-scale mining operations in the 1960s [13]. This process involves treatment of metallic gold with mercury to form an amalgam containing 40–80% mercury by mass [21], which is then heated to vapourise the toxic mercury and recover the gold content. Despite the well-known dangers of this method, amalgamation is still used in present-day artisanal gold mining. It is estimated that between 410 and 1400 tonnes of mercury vapour is emitted this way each year, accounting for 37% of global mercury emissions [21]. This telling statistic underscores the toxicity of artisanal gold mining, and how the gold sector is reported to generate more pollution that the production of all the other elements in the periodic table (Figure 1) [22].

Due to these environmental and safety concerns, alternative hydrometallurgical processes based on less dangerous and polluting reagents have increasingly been explored. The main approach employs *S*-donor complexing agents operating in an oxidising environment. Despite some unresolved drawbacks, mainly related to ligand-consuming side reactions, which currently limit their wider application in mining, notable success has been achieved. For example, the use of acidic thiourea or basic thiosulfate leaching in the presence of oxidants (mainly Fe^3+^ ions) has been demonstrated to be the most promising alternative to cyanidation for gold reclamation from ore [23,24].

The severity of the environmental impacts caused by gold mining can also be attributed to the low grade of ore used for gold production compared to that used in the extraction of other metals. Haque et al. [25] reported that even though high grade ore can contain up to 40 g gold per tonne, the world mean ore grade for gold contains just 3–4 g of gold per tonne, with low grade ores containing as little as 0.5–1.5 g gold per tonne. This is a fraction of what can typically be mined from one tonne of copper ore (6–10 kg) [26], or ores of other precious metals such as silver (40–400 g) [27]. Due to the inherent scarcity of gold, a much greater quantity of ore material needs to be processed in the mining and comminution stages, which translates to a greater environmental impact [28,29]. 

Despite the many properties of gold, which make it the metal of choice for a wide range of applications, its high and volatile price, as well as the negative environmental impact of its production, as outlined above, discourage its use on a large scale. The possibility of using secondary raw materials as a source of gold will help to ensure the future use of this metal in high-value applications.

### 2.2. Gold from Secondary Sources

**Figure 2:**
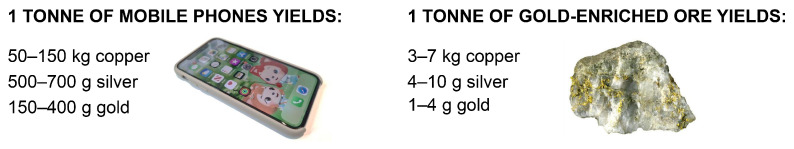
Comparison of the amount of various metals yielded from recycled mobile phones compared to primary ore [35].

Gold recycling currently contributes a sizeable portion of the global gold supply (around 30%) [30]. However, high-value gold source materials (e.g., gold jewellery, bars and coins) currently account for roughly 90% of the total recycled gold supply with only 10% coming from industrial sources, such as WEEE. Life cycle assessments often reveal that the environmental consequences associated with gold production would be reduced by the ‘urban mining’ of secondary waste sources [31,32]. A significant reduction in CO_2_ emissions can be attributed to the recycling of gold in this more concentrated and more readily available form than in natural ore [33]. Unfortunately, the recovery of gold from WEEE is far more difficult than from high-value secondary sources such as jewellery scrap. As a result, just 2% of global WEEE is treated for gold recycling. Nevertheless, the growing volume of WEEE and its composition of various valuable metals offer great opportunities for the recycling of these end-of-life products. It is estimated that one tonne of discarded mobile phones can provide 150–400 g of gold, in contrast to just 1–4 g of gold from a similar tonnage of ore (Figure 2) [34].

Other metals such as silver and copper can similarly be recovered in much higher yield, therefore indicating the huge material gain available from such WEEE. The global volume of WEEE being produced annually stands at over 50 million tonnes (53.6 Mt in 2019) [22,36], and this figure is expected to rise to 100 million tonnes by 2025 [12]. Despite such a huge amount of waste being generated each year, only 20% is formally recycled, with the vast majority being either sent to landfill or incinerated [22]. Consequently, governments across the world have actively been introducing WEEE directives in their countries to legislate for the proper disposal and treatment of WEEE [37,38].

Pyrometallurgical approaches are the most widely used at an industrial level for the gold refining step and for the recovery of primary feed concentrates and secondary sources. Currently, most WEEE is treated using the pyrometallurgical processes established for primary feed concentrates and/or high-value precious metal waste (mainly comprising jewellery and residual copper scraps) in large-scale state-of-the-art recovery plants [33].

**Figure 3:**
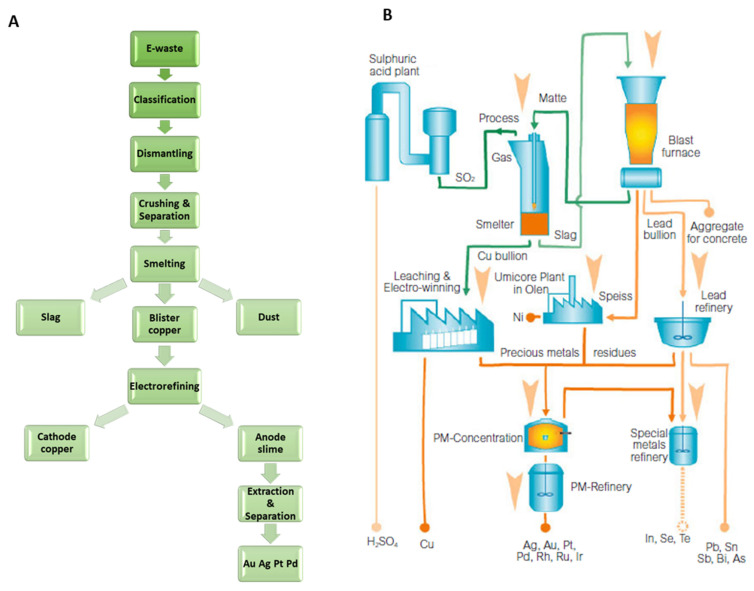
Flowsheets of (**A**) precious metals being recycled from WEEE by a pyrometallurgical process, reproduced with permission from [39] and (**B**) simplified plan of the Umicore integrated smelter-refinery plant, reproduced with permission from [40].

A typical pyrometallurgical process commences with pre-treatment of the WEEE, involving classification, dismantling, crushing, and separation. Small amounts of this pre-treated waste are then added to the mainstream material (e.g., 14% of overall throughput in the case of the Horne Smelter) and smelted to obtain a precious metal copper-bearing bullion (Figure 3A) [39,41]. Subsequent electrorefining produces high purity copper at the cathode, and an anode sludge residue, which can be further refined through hydrometallurgical processes to recover precious metals such as Ag, Au, Pt, Pd, Rh, Ru, and Ir. Smelting also provides oxidised base metals which are captured in a silica-based slag, while the plastic components are incinerated. One example is the integrated smelter and refinery plant of Umicore in Hoboken, Belgium (Figure 3B), which reports recovery precious metals rates of over 95% with a CO_2_ abatement potential of 1 million tonnes (Mt) compared to primary metal production (estimated to cause 1.3 Mt of CO_2_ emissions) [31]. Nevertheless, pyrometallurgical processes are highly energy-intensive and require large capital investments to construct their state-of-the-art recovery facilities. Furthermore, pyrolytic processes applied to complex materials such as WEEE that contain large amount of plastics and resins produce toxic gaseous (dioxins) and carbonaceous small-particle emissions if not well managed. As an alternative, hydrometallurgical processes represent a versatile tool for designing cheaper, less energy-intensive and selective processes for metal recovery, which in turn simplifies the production of high purity metals by reducing the number of recovery steps [42].

A typical hydrometallurgical process usually consists of 3 main steps: (1) a mechanical pre-treatment of waste; (2) leaching of metals using a suitable lixiviant; and (3) purification and recovery of metals from the leaching solution [43]. Mechanical pre-treatments expose the metal to the chemical etching process and often involve costly but unavoidable disassembling, shredding, and separation phases. Since metals such as gold are typically present in their elemental form, an oxidative leaching process is required for the effective recovery of the precious metal [33]. Depending on the lixiviant system used, different types of gold complexes can be generated in solution. These can be anionic or cationic, depending on the system used, and the solution itself can be acidic, neutral or alkaline (Table 1). 

The first two entries in Table 1 represent the most widely used systems with *aqua regia* being the most used leaching agent (even more than cyanidation) for precious metal refining in formal and informal gold recovery processes. This makes HAuCl_4_ solutions the most commonly produced from metal reclamation. Several reviews have covered the advantages and disadvantages of each lixiviant [42,45], reflecting the fact that each lixiviant system has disadvantages, such as the environmental consequences of cyanide and *aqua regia* chloride leaching. Leaching with thiosulfate and thiourea is more environmentally friendly but these approaches suffer from the high reagent consumption used during the process. As a result, other leaching systems which use milder, more selective and environmentally viable lixiviants and conditions have been proposed and patented recently. These typically exploit the use of safe and recyclable ‘soft’ donor ligands in oxidising environments for the gold recovery phase [46]. The selectivity of this alternative approach is a key point. When not provided by pre-treatments and leaching, the selectivity in a system needs to be integrated into the recovery phase by electrowinning or cementation, or in an intermediate selective concentration/separation phase [33]. Besides the well-known and widely employed adsorption on activated carbon, selective solid-liquid extraction and precipitation with innovative and/or bioderived materials have been proposed and give rise to gold-enriched solid materials. Besides the selective absorption of metal complexes onto solid-state supported materials, by far the most popular technique investigated is solvent extraction. Several extractants and new solvents, which are greener with respect to conventional volatile organic solvents, have been developed which provide excellent selectivity [31,39,42,47,48,49,50,51,52].

Hydrometallurgical approaches lead to the formation of gold compounds, typically obtained as metal complexes, which can be isolated from the leaching or extraction solutions as well as from solid extractants or precipitates. In a typical recovery process, these compounds or materials would need to undergo further costly and often environmentally problematic refining steps to integrate into existing markets and applications for precious metals. A possible alternative is to use the compounds or gold-enriched recovery materials obtained from the process directly in high-value applications, such as catalysis.

### 2.3. The Role of Gold in Catalysis

Gold is now established firmly as an important metal in both homogeneous and heterogeneous catalysis despite its historical chemical inertness, which led to it being overlooked for application in catalysis originally. Indeed, the first report of a homogeneous gold-catalysed reaction emerged only in 1976. In that year, Thomas and co-workers reported conversions of alkynes to ketones using tetrachloroauric acid with yields up to 38% [53]. The field of homogeneous gold catalysis remained essentially dormant throughout the 1980s and 1990s, with Dyker’s review in 2000 citing as few as 13 reactions catalysed by gold under homogeneous conditions [54]. However, over the past two decades, the scope of homogeneous gold catalysis has expanded significantly, with reports now describing cross-coupling reactions, C-H activation and photoredox catalysis [55,56,57,58,59,60,61]. Despite the advances in homogeneous gold catalysis, the use of heterogeneous supported catalysts has dominated industrial applications of gold catalysts, mainly in the synthesis of bulk chemicals [62]. 

It is worth noting that gold has a reputation for promoting organic transformations for which no other metal catalysts have been identified. For example, gold offers a catalytic replacement for common stoichiometric Lewis acid systems, such as FeCl_3_/BF_3_, with catalytic inter- and intramolecular activation of alkynes, alkenes and carbonyl compounds being particularly widely studied [63,64,65,66,67]. For this reason, the direct use of gold recovered from WEEE as a catalyst, as opposed to lighter transition metal and main group alternatives, offers a more sustainable source of gold than primary mining, while supporting the continued use of gold catalysis. 

### 2.4. Direct Application of Recovered Gold in Catalysis

Due to the unique properties of gold and the interest in valorising recovery processes, the use in catalysis of gold compounds and materials obtained as recovered products of hydrometallurgy has been selected here as a case study of an innovative circular economy approach.

**Figure 4:**
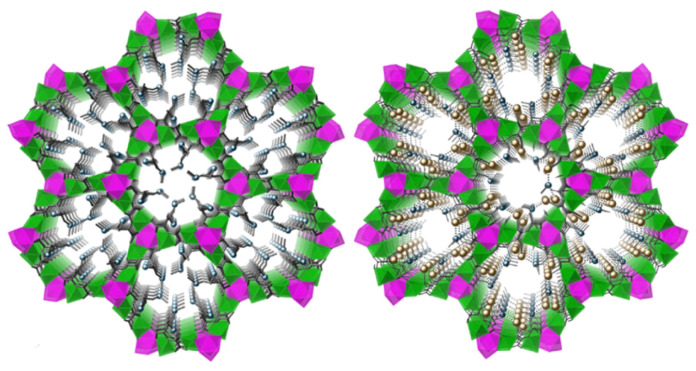
Structures of a MOF based on Ca^2+^ and Cu^2+^ employed for gold recovery (**left**), and its gold recovery product (**right**). Reproduced with permission from [68].

A recent example of gold recovery followed by direct use of the recycled gold material in catalysis has been reported by Pardo et al. [68]. In this work, a MOF based on divalent Ca and Cu_6_ units was prepared containing channels decorated with thio-alkyl chains derived from L-methionine as a selective solid extractant for gold from leaching solutions. Initially, Au^3+^ and Au^+^ species were recovered from a solution of either AuCl_3_ or AuCl in water-methanol mixtures. In the case of AuCl_3_, a gold-loaded MOF with an Au^3+^:MOF molar ratio of 3:1 was obtained. In the case of AuCl, recovery led to a 2:1 ratio of Au^+^ and MOF. The selectivity of the recovery process towards gold was investigated by soaking the MOF in an equimolar solution of gold ions along with [Pd(NH_3_)_4_]Cl_2_, NiCl_2_, CuCl_2_, ZnCl_2_, and AlCl_3_. Analysis using inductively coupled plasma atomic emission spectroscopy (ICP-AES) revealed that the recovery process was completely selective for gold with a maximum loading being achieved after 60 min. Crystallographic analysis by X-ray diffraction illustrated that the highly flexible nature of the thio-alkyl chains enabled metal binding to be achieved without distorting the porous network (Figure 4).

**Figure 5:**
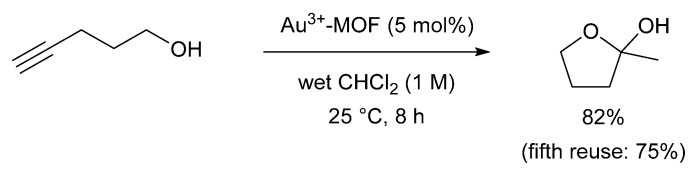
Cyclisation of 4-pentyn-1-ol catalysed by recovered gold, Reproduced with permission from [68].

This high three-dimensional stability, even after gold capture, represented an unprecedented adsorption capacity (15–20 wt. %) for the MOF material. Consequently, the group explored the catalytic activity of the Au^3+^-loaded MOF (Au^3+^-MOF) in the cyclisation of 4-pentyn-1-ol (Figure 5).

The reaction catalysed by Au^3+^-MOF led to an 82% yield, compared to 60% provided by AuCl under identical conditions. In situ UV-Vis spectroscopy of the reaction mixture using AuCl as the catalyst revealed a new adsorption band at ~500 nm, which was attributed to the formation of catalytically inactive Au^0^ nanoparticles that were not observed in the reaction catalysed by Au^3+^-MOF. The stability of Au-MOF was illustrated further in recycling experiments, where the gold-loaded MOF was reused 5 times in the cyclisation of 4-pentyn-1-ol, with only a small drop in yield being observed after the 5th cycle (82% to 75%).

**Figure 6:**
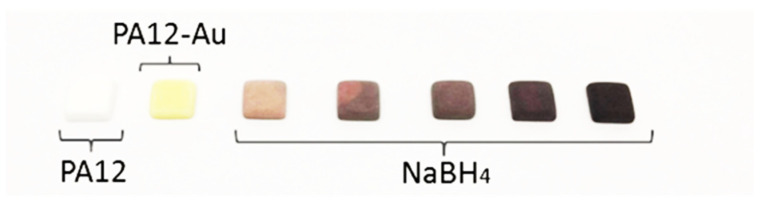
Polyamide adsorbent used for gold recovery and catalysis. PA12 is the unused adsorbent, PA12-Au is the adsorbent after gold recovery. NaBH_4_ indicates the Au^0^ loaded PA12 after reduction by NaBH_4_. Reproduced with permission from [69,70].

Heterogeneous adsorbents have also been used for gold recovery by Haukka and colleagues, who employed a highly porous polyamide (polyamide-12/Nylon-12, PA12) adsorbent to recover gold from the leachate from waste printed circuit boards (PCB) treated with *aqua regia* (Figure 6) [69]. The adsorbent was 3D printed using selective laser sintering and was reported to selectively recover gold from an acidic PCB leachate containing a mixture of other transition and precious metals, including platinum and palladium. Crucially, only 2% tin (from solder) was extracted alongside 78% recovery of gold. The 3D printing process also enabled a variety of highly porous shapes of adsorbent to be produced, including cylindrical columns for potential applications in-flow [70]. Following gold recovery from WEEE, the gold-loaded PA12 adsorbent was reduced using NaBH_4_, yielding PA12-supported Au^0^ nanoparticles with diameters between 14 and 32 nm.

**Figure 7:**
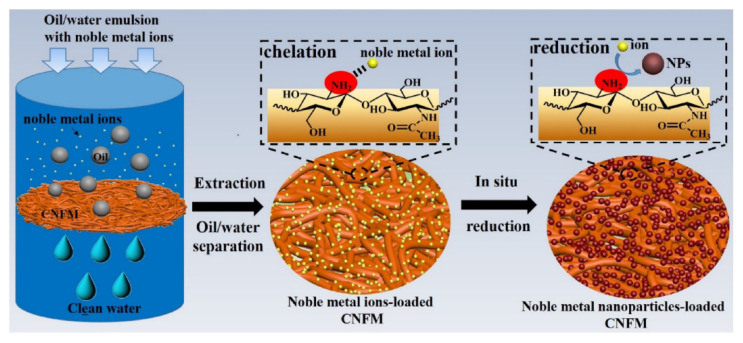
Chitin nanofibrous membrane employed for gold recovery and catalysis. Reproduced with permission from [71].

The resulting nanoparticles were shown to effectively catalyse the reduction of 4-nitrophenol to 4-aminophenol, providing quantitative conversion after 2 h over 10 reuse cycles. The same reaction was the focus of a study using catalysis by recovered gold by Liu and colleagues in 2019 [71]. This report used chitin, a popular adsorbent and catalyst support derived from waste bio-cellulose [72,73,74,75], as a nanofibrous membrane to recover gold from an organic/aqueous emulsion (Figure 7). Good uptake capacities for Au^3+^ (~248 mg·g^−1^), Ag^+^ (~136.5 mg·g^−1^), Pt^4+^ (~217.8 mg·g^−1^) and Pd^2+^ (197.8 mg·g^−1^) were reported, although the selectivity for Au^3+^ in the presence of other metals was not explored. The trivalent gold ions loaded onto the chitin membrane were then reduced to Au^0^ nanoparticles of around 5 nm in diameter after heating at 90 °C for 30 min without the need for an additional reductant. The amine groups present in chitin were proposed to be crucial for the reduction of Au^3+^, as no reduction was observed when a TEMPO/NaClO oxidised chitin sample was used, although the authors did not comment on the possibility of other competing factors, such as alcohol oxidation. In addition, comparisons of the XPS spectra for Au^3+^ and Au^0^ chitin nanofibres revealed differences in the nitrogen binding energies; the carbon and oxygen environments were essentially unchanged in the two samples.

The group went on to explore the catalytic activity of their Au^0^ chitin nanofibres in the reduction of 4-nitrophenol. Quantitative conversion to 4-aminophenol was observed at room temperature by in situ UV-Vis spectroscopy; however, further details on the reaction duration, for example, were not provided.

Despite the relative scarcity of examples to date, these investigations have revealed very promising catalytic properties for the catalysts obtained from gold recovery, paving the way for further studies in the field.

## 3. Palladium

### 3.1. Primary Production of Palladium

Primary sources of palladium supply around 69% of global demand for the metal [5] Palladium mining is typically performed through conventional open-pit or underground techniques and thus suffers from many of the hazards previously described for gold mining [76]. Once extracted, the ore is ground and processed by froth flotation to produce a concentrate rich in precious metals, typically the Platinum-Group Metals (PGMs: Pt, Pd, Rh, Ru, Ir, Os). The concentrate is then smelted in electric-arc or blast furnaces at temperatures >1000 °C, before being purified at a refinery for precious metals. The scarcity of these resources mainly localized in South Africa and Russia (supplying respectively the 37% and 42% of the palladium produced in 2019) [77], the energy-intensive processes required for the production of these metals, compounded by the unrelenting demand from the automotive industry, have culminated in the election of palladium among global scale critical raw materials and record prices for the metal (GBP 19 per gram in 2016 to GBP 85 per gram in 2021) [78].

Many of the environmental issues surrounding the supply of gold described in the previous section also apply to palladium, including the negative impacts of mining on local water supply, housing displacement and biodiversity [3,76,79]. In addition, the presence of other PGMs in natural ore supplies, close to palladium in terms of chemical properties, means that refining palladium ore is a challenging energy- and resource-intensive process. An extensive analysis of the environmental consequences of palladium mining and refining was reported by Nuss and Eckelman in 2014, who estimated that 3880 kg of CO_2_ equivalents are emitted per kilogram of palladium produced [2]. In addition, the process uses *aqua regia* to dissolve palladium, generating toxic NO_x_ and Cl_2_ gases, harming workers and local biodiversity, as well as producing large quantities of contaminated waste water [3,76,79]. It is, therefore, imperative that changes are made in the way that palladium is produced and consumed if the applications of this metal are to become sustainable.

### 3.2. Palladium from Secondary Sources

Consumption of palladium is dominated (~90%) by its use in three-way catalytic converters (TWCs) and WEEE [80]. The inherently short lifetime (around 10 years) of these materials has resulted in an accumulation of palladium-rich waste. Similar to gold, considerably more palladium is present in TWCs (2000 g/tonne) and WEEE (300 g/tonne) than is present in natural ore (<10 g/tonne) and thus these waste streams offer a ‘closed-loop’, sustainable source of palladium metal [81].

Unfortunately, existing recovery technologies use a combination of resource-intensive pyro- and hydrometallurgy. For example, the process used by Johnson Matthey involves smelting the spent catalyst at 1500–1650 °C in a crucible containing a molten collector metal, such as copper or iron [82,83]. The resulting molten slag settles under gravity and the separated PGMs are tapped off and refined. Recovery of PGMs above 95% is achieved; however, recycling comes at a significant cost in resources. This was highlighted by Ramnäs and Amatayakul in their life cycle assessment of TWCs, in which they reported that although the recycling of TWCs reduces solid waste production and provides an alternative to the finite natural supply, other damaging environmental impacts, such as consumption of energy resources and emissions (both air and water) are increased [84]. In particular, the authors highlighted that increased crude oil and natural gas consumption during TWC recycling outweighs the reduction in the use of coal during primary production, thus highlighting the need for more sustainable methods for recycling waste TWCs.

Research efforts investigating more sustainable methods for recovering palladium have primarily focused on hydrometallurgy, as it is typically less energy-intensive and offers greater potential for selectivity. The wide range of strategies employed in hydrometallurgical palladium recovery have been reviewed [48,49,50,51,85,86]. Consequently, the remainder of this overview will focus on examples reporting a direct application of the recovered palladium in catalysis.

### 3.3. The Role of Palladium in Catalysis

Beside the cited large application in heterogeneous oxidative catalysis in automotive TWCs, palladium has become an indispensable metal in modern synthetic chemistry, with applications ranging from bench-scale research to industrial scale production [87,88,89,90,91]. A wide range of intermediates in the synthesis of pharmaceuticals are produced by a palladium catalysed cross-coupling reactions, with the Suzuki–Miyaura coupling being the most prevalent. Similarly, palladium catalysis has been applied in the industrial scale synthesis of agrochemicals [87,92,93,94]. Due to this crucial role played by palladium in catalysis, the environmental and economic costs associated with primary palladium supply has significant implications for the chemical industry. To tackle these challenges, several different strategies have emerged. These include catalysts that function at low-loadings [95], catalysts capable of being reused multiple times [96], and the substitution of palladium by first-row transition metals [6,7,8]. Despite providing significant improvements over traditional palladium usage, low-loading and reusable catalysts still depend on a finite natural supply of palladium. Although the use of more abundant transition metal alternatives (such as Ni, Cu, Fe, Co) is an exciting and worthwhile area of research, palladium continues to be used very widely for several reasons, despite the economic and environmental issues surrounding primary palladium supply. For example, divalent palladium precatalysts are stable towards moisture and oxygen in contrast to many catalysts based on lighter transition metals [9,10,11,90]. Moreover, Pd^2+^ precatalysts react with a range of *hard* and *soft* organometallic reagents and are readily reduced in situ to yield an active Pd^0^ species. The corresponding precatalysts based on lighter transition metals often require more reactive organometallic reagents (e.g., Grignards) and/or the addition of sacrificial reductants, which can impact on functional group tolerance [10,11]. Finally, palladium catalysts operate under milder conditions, in greener solvents and at lower catalyst loadings, which can diminish the economic and environmental benefits associated with lighter transition metal alternatives [11]. Palladium catalysis is also firmly entrenched in the armoury of synthetic chemists, providing substantial inertia towards the idea of using alternative approaches based on earth-abundant metals. Consequently, researchers have begun exploring the possibility of employing palladium recovered from spent TWCs or WEEE directly in catalysis. Using material recovered from such waste offers a more sustainable source of catalysts while still benefiting from the superior catalytic properties of palladium.

### 3.4. Direct Application of Recovered Palladium in Catalysis

**Figure 8:**
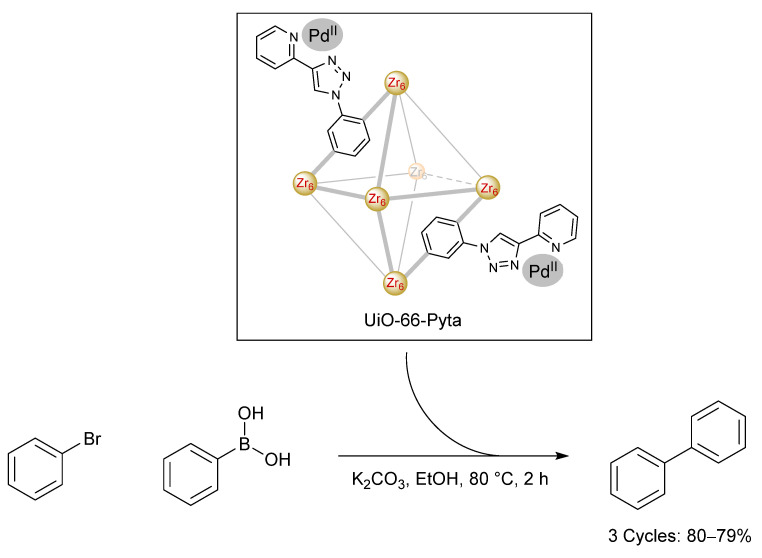
Palladium-loaded UiO-55-Pyta MOF employed as a Suzuki–Miyaura catalyst [97].

Owing to the popularity of acidic leaching of palladium from spent TWCs and WEEE, several groups have adopted these intermediate acidic waste streams as sources of palladium for catalysis. One such example was reported by García et al., who used a zirconium MOF to selectively extract palladium from an acidic solution containing Zn^2+^, Pb^2+^, Fe^3+^, Mn^3+^, Ni^2+^, Co^3+^, Al^3+^, Cu^2+^ and Ag^+^ ions (Figure 8) [97]. The MOF (UiO-66-Pyta) was functionalised with a pyridyl-triazole ligand, which was shown to significantly improve the palladium capacity (97% recovery, 294 mg·g^−1^) and adsorption rate (5 min). The MOF was also reused after a relatively mild desorption step (1 M nitric acid) and was recycled 5 times, with a small drop in recovery yield (97% to 82%) being reported after the 5th reuse. The group also explored the activity of their palladium-loaded MOF as a Suzuki–Miyaura catalyst.

The palladium-loaded MOF exhibited good catalytic activity and was reused 3 times following recovery of the catalyst by centrifugation. Scanning electron microscope (SEM) imaging of the recovered palladium-loaded MOF following catalysis illustrated that the catalyst had not undergone any observable structural changes, while ICP-AES analysis of a reaction solution revealed only negligible amounts of palladium leaching. When compared to conventional extractants, the MOF exhibited excellent extraction capacity and rates. Further work is needed to explore the possibility of recovering palladium from real or model waste streams.

**Figure 9:**
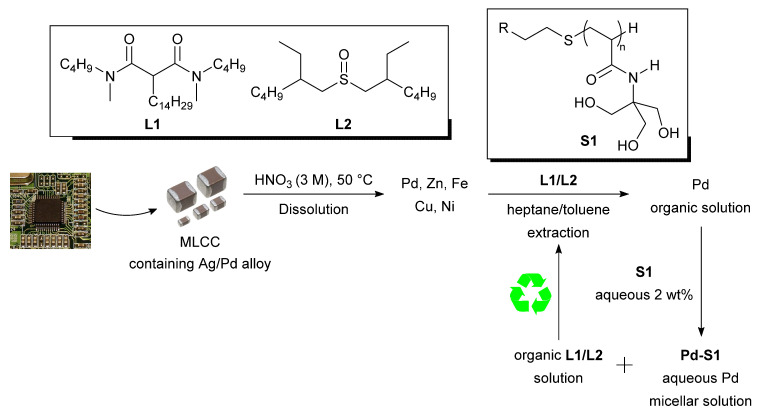
Micellar back-extraction of recovered palladium [98]. Reproduced with permission [98].

In a recent report, Bourgeois et al. described a process for recovering palladium as a catalytic micellar solution from multi-layer ceramic capacitors (MLCC) recovered from WEEE. The group employed their previously reported recovery process, which involved selective dissolution of silver and palladium from the gold present using a heated (50 °C) 3M nitric acid solution [98]. After leaching, palladium was selectively extracted using one of two organic extractants tested (**L1** and **L2**), followed by back-extraction with a surfactant based on tris-(hydroxymethyl)acrylamide (**S1**) (Figure 9). The recovery process was found to be selective for palladium, provided up to 97% recovery yield after the extraction/back-extraction steps, and the organic phase containing the extractant could be reused (limiting waste production to 10% by volume of the organic phase).

The group then explored the catalytic activity of their micellar palladium solution. Initially, **S1** was loaded with Pd(OAc)_2_ and used to catalyse the Suzuki–Miyaura coupling of *m*-bromoanisole and phenyl boronic acid in the presence of P(^t^Bu)_3_. The results were then compared to a micellar solution containing recovered palladium (Table 2).

The micellar solution exhibited good catalytic activity, especially given the mild conditions, although the solution containing recovered palladium was slightly less active than Pd(OAc)_2_.

**Figure 10:**
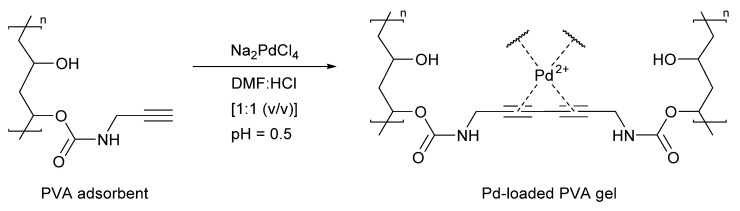
Palladium recovery enabled by a Glaser coupling to yield a cross-linked gel, Reproduced with permission from [99].

In 2019, Zhao et al. described a novel strategy for palladium recovery, wherein selective recovery was achieved by exploiting the metals unique catalytic activity [99]. The group developed a polyvinyl alcohol (PVA) adsorbent containing alkyne side chains. The PVA adsorbent was treated with an acidic [PdCl_4_]^−^ solution, which promoted the cross-linking of the alkyne chains and subsequently formed a porous PVA-gel (Figure 10). Divalent palladium ions were consequently trapped in the pore network of the PVA-gel, while any other metals present were washed away by immersing the gel in water. The technique provided 79% palladium recovery (152.37 mg·g^−1^) from a mixed metal solution (Pd, Al, Ni, Zn, Fe, Co), with only Al^3+^ being co-adsorbed in any significant quantity (~10%). The group also illustrated that a model Glaser coupling could only be catalysed by the material containing Pd^2+^ ions. This led to the authors suggesting that the selective entrapment of palladium was a direct result of the metals involvement in the cross-linking of the PVA-gel. It is worth noting that a preformed PVA-gel was not investigated for the recovery of palladium. It would be interesting to know also whether palladium could be recovered from either a real or model waste stream.

**Figure 11:**
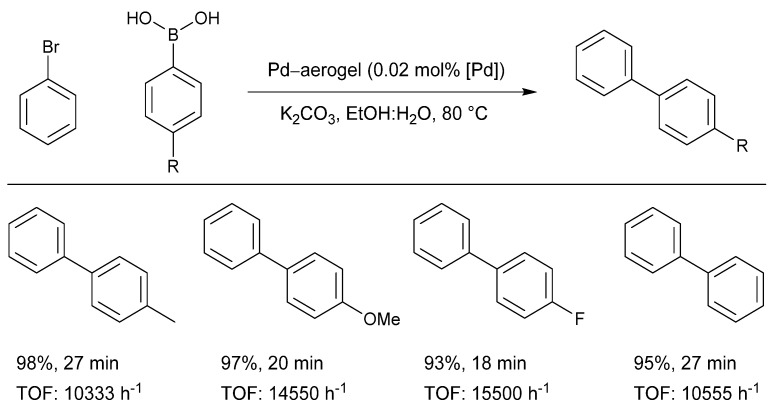
Activity of a palladium-loaded aerogel as a Suzuki–Miyaura catalyst, Reproduced with permission from [99].

The palladium-containing gel was reduced by NaBH_4_, followed by freeze drying, yielding palladium nanoparticles of around 1.9 nm in diameter loaded onto a PVA aerogel. The group subsequently investigated the catalytic activity of the Pd-loaded aerogel in the Suzuki–Miyaura coupling of a series of aryl boronic acids and bromobenzene. The nanoparticles functioned at a low catalyst loading (0.02 mol%) under environmentally benign conditions, providing excellent turn over frequencies (TOFs) (Figure 11).

The Pd-aerogel could also be recovered and reused at the end of the reaction with no significant drop in catalytic activity being observed after 6 reuse cycles.

**Figure 12:**
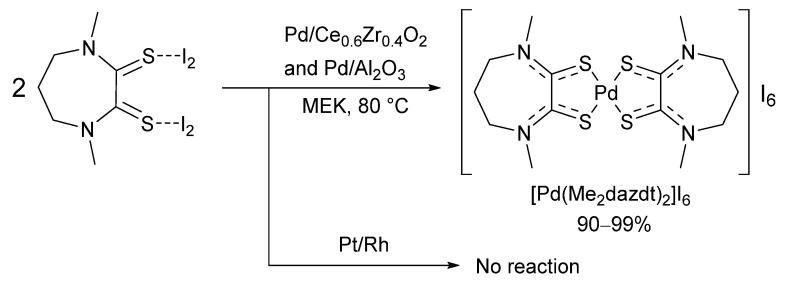
Recovery of palladium using Me_2_dazdt·2I_2_, Reproduced with permission from [100,102].

The significant environmental and practical costs associated with the harsh conditions of metal leaching solutions such as *aqua regia* have prompted researchers to investigate alternative, safer reagents that work under milder conditions. An innovative approach was proposed in the 1990s by Deplano and co-workers, based on the use of dithiooxamide/dihalogen charge transfer compounds in organic solvents with noble metals [100]. The bis-diiodine adduct of *N,N′*-dimethylperhydrodiazepine-2,3-dithione (Me_2_dazdt 2I_2_) combines complexing and oxidising properties within the same molecule. Acetone solutions of Me_2_dazdt 2I_2_ were shown to be very effective in dissolving noble metals such as gold [101] and palladium [102] under mild conditions and their use in the selective leaching of palladium from spent TWC was patented [100,101,102,103]. The group reported 90–99% palladium recovery from a model catalytic converter support under mild, non-acidic conditions (Figure 12). 

The adduct, Me_2_dazdt·2I_2_, was found to be completely selective for palladium, rendering the technique particularly attractive for recovering palladium from spent TWCs that typically also contain platinum and rhodium [104]. Despite the efficacy of the process for recovering palladium, the poor atom-economy of Lawesson’s reagent used in the synthesis of Me_2_dazdt, as well as the lack of commercial availability of the dithiooxamide reagent, hinders the industrial viability of this process. As a result, the same group has explored, with expert research groups in the field, direct valorisation of the recovery product, both as a homo- and heterogeneous catalyst [105,106,107]. Finding a direct use for the palladium recovery product [Pd(Me_2_dazdt)_2_]I_6_ in this way helps improve the economic viability of the process and provides synthetic chemists with catalysts derived from a secondary source of palladium. In an early example, an acetone solution of [Pd(Me_2_dazdt)_2_]I_6_ was used to prepare a Pd(0.5%)-TiO_2_-based photo-reforming catalyst by an impregnation/photo-reduction technique [105]. The prepared photo-catalyst showed an 80% enhancement in H_2_-production from 1M methanol and glycerol aqueous solutions compared to reference photo-catalysts obtained from conventional Pd(NO_3_)_2_ and PdCl_2_. This increased activity was ascribed to the higher metal dispersion and reduced Pd^0^ particle size achieved using a recovered coordination compound instead of commercially available palladium salts as suitable precursors. In a second example, [Pd(Me_2_dazdt)_2_]I_6_ was used to catalyse the oxidative C-H functionalisation of benzo[*h*]quinoline and 8-methylquinoline (Table 3) [106].

**Figure 13:**
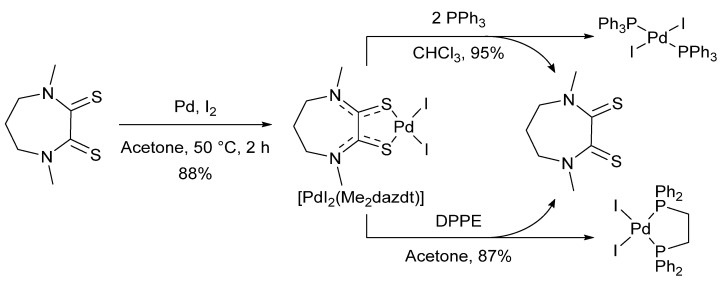
Expanded recovery products derived from Me_2_dazdt·I_2_, Reproduced with permission from [107].

Compared to Sanford’s original report using palladium(II) acetate as the pre-catalyst [108], [Pd(Me_2_dazdt)_2_]I_6_ provided similar yields under considerably milder reaction conditions. It was later shown that a related recovery product, [PdI_2_(Me_2_dazdt)], could be generated from palladium metal by changing the Me_2_dazdt:I_2_:Pd ratio to an equimolar ratio (Figure 13). Similar recovery yields were obtained as for Me_2_dazdt·2I_2_ (albeit under slightly more forcing conditions), which represents a considerable reduction in material consumption. In addition, [PdI_2_(Me_2_dazdt)] could be further functionalised through ligand exchange with phosphines to yield additional catalytically useful products, while recovering the Me_2_dazdt ligand (Figure 13) [107].

**Figure 14:**
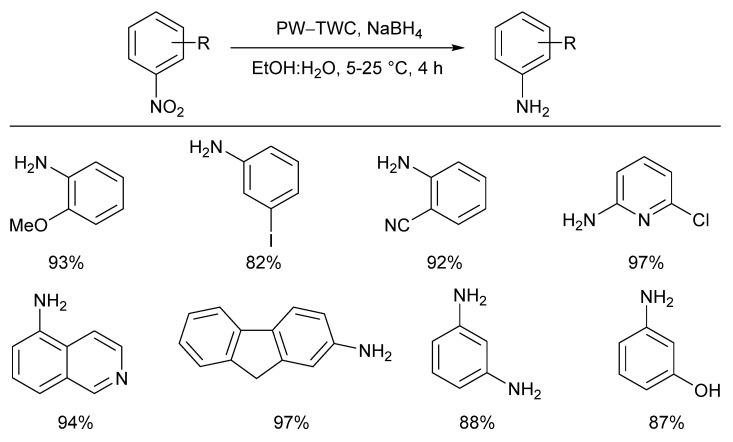
Application of three-way catalyst (PW-TWC) material applied directly in milled form as a catalyst for the reduction of nitroarenes, Reproduced with permission from [109].

To date, these examples of palladium catalysts have been recovered either from acidic leach solutions, or directly from the solid waste using non-acidic oxidative dissolution. Several groups have explored using scrap TWCs directly as a heterogeneous catalyst, bypassing the need for any leaching steps. Avoiding the need for leaching reagents in this way provides a clear environmental advantage. However, since there are other valuable base and precious metals present in spent TWCs that could be recovered during leaching, the economic viability of this approach remains unclear. One example of this strategy was described in 2015 by Genc and co-workers, who used a powdered waste TWC sample (PW-TWC) to promote the reduction of a broad range of nitroarenes [109]. The PW-TWC sample was subjected to multiple washes with acetone and water before being dried at 400 °C. The PW-TWC sample was then analysed with XRF analysis, revealing the presence of a mixture of transition metals that could have been responsible for the catalytic activity displayed, including Pt (0.491 wt.%) and Pd (0.115 Wt.%). A broad scope of 20 different nitroarenes were reduced by PW-TWC in the presence of NaBH_4_ (Figure 14).

The reusability of the PW-TWC catalyst was also investigated in the reduction of nitrobenzene with no loss in activity being observed after 6 reuse cycles.

**Figure 15:**
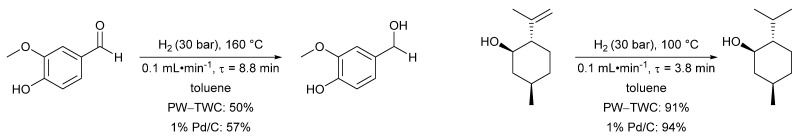
Application of powdered waste three-way catalyst (PW–TWC) materials as in-flow alkene hydrogenation catalysts. Residence time (τ = catalyst cartridge volume/flow rate), Reproduced with permission from [110].

A more recent example of PW-TWC being employed as a catalyst was reported by Luque et al. [110], in which the group used milled PW-TWC material as a heterogeneous hydrogenation catalyst. The PW-TWC sample was initially washed with water, ethanol and acetone, and subsequently dried at 100 °C. Thermogravimetric analysis (TGA) revealed the importance of this washing procedure, as an unwashed sample exhibited a mass loss of around 20%, attributed to organic impurities, which was not observed for the washed PW-TWC material. The washed PW-TWC sample was employed as an in-flow hydrogenation catalyst to investigate a few different substrates with comparisons made to a commercial Pd/C catalyst (Figure 15).

**Figure 16:**
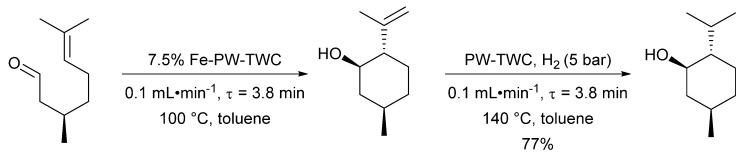
Application of PW-TWC material as an in-flow tandem cyclisation/hydrogenation catalyst. Residence time (τ = catalyst cartridge volume/flow rate, Reproduced with permission from [111].

A decrease in yield for the hydrogenation of (−)-isopulegol from 94% to 85% was recorded after 2 h with no additional subsequent loss in activity being observed. The loss in activity was attributed to the adsorption of reagents on the catalyst surface and a toluene wash cycle was sufficient to restore the original catalyst activity. In addition, scanning transmission electron microscope high-angle annular dark-field (STEM-HAADF) imaging of the PW-TWC indicated that neither leaching nor aggregation of palladium had occurred after 8 h of reaction. Additional research by the Luque group has expanded on their work on PW-TWC, applying material derived from scrap to the continuous in-flow synthesis of menthol from citronellal [111]. The PW-TWC sample was employed both as a solid support for iron oxide nanoparticles (Fe-PW-TWC) to catalyse the cyclisation of citronellal and as a catalyst for the hydrogenation of (−)-isopulegol (Figure 16).

When compared to a commercial Fe/SiO_2_ catalyst, the Fe-PW-TWC material resulted in improved yields. The great stability of the powdered waste TWC sample was again illustrated, as no drop in activity was observed until after 7 h of reaction, and no significant catalyst leaching was observed on analysis of the product with inductively coupled plasma mass spectrometry (ICP-MS).

## 4. Conclusions and Future Outlook

Over the past decade, significant contributions have been made to uncover more sustainable sources of gold and palladium. In particular, hydrometallurgical approaches for metal recovery offer improved selectivity and reduced energy consumption relative to traditional pyrometallurgical approaches. However, the significant environmental consequences associated with reagents such as cyanide and *aqua regia* still impede the environmental benefits of hydrometallurgical recovery. The use of soft donor- and polyhalide-based lixiviants has emerged as a promising replacement for *aqua regia*. Moreover, several groups have adopted these approaches and have used recovery products to provide synthetic chemists with precious metal catalysts derived from secondary sources. Advances have been made in particular in hydrogenation reactions that use recovered metals; however, additional research is needed to expand the scope of recovery products to the most widely used C-C and C-N bond-forming reactions. In the future, the application of recovery products in catalysis could be especially pertinent for those lixiviants that rely on harsh conditions or additional reagents (e.g., acidified thiourea) for metal stripping.

Given that waste streams originating from WEEE and TWCs often contain a mixture of base and precious metals, the importance of selectivity in any recovery process cannot be ignored. Despite this, the selectivity of many recovery processes for the desired metal is often not explored. Even for those examples that do explore selectivity, many reports overlook the selectivity between different precious metals—an observation that is likely to relate to the difficulties often encountered in separating precious metals. Future research in the area is thus encouraged to explore selectivity for the desired metal using a model or real waste stream. Not only would this prove more persuasive to those looking to adopt a recovery process on an industrial scale, but the selectivity data would also provide information to guide future research toward more selective recovery techniques.

Finally, in line with worldwide recommendations on the implementation of smart and sustainable circular economy models, process design should aim to harmonise the recycling methods with the properties of the recovered products for specific applications. Hydrometallurgy represents a very versatile tool that can be harnessed to provide specific functional materials as recovery products, ready to meet the requirements for application in value-added manufacturing processes.

## Figures and Tables

**Table 1:** Summary of the different lixiviant systems for gold extraction from WEEE [33,44].

Lixiviant	Ligand	Oxidant	Gold Complex in Solution	Leaching Conditions
Cyanide	CN^−^	O_2_	[Au(CN)_2_]^−^	E°: −0.67 V pH > 10, 85 °C
*Aqua regia*	Cl^−^	HNO_3_	[AuCl_4_]^−^	E°: 0.96 V pH > 8–11, > 50°C
Thiosulfate	S_2_O_3_^2-^	O_2_ or Cu^2+^	[Au(S_2_O_3_)_2_]^3−^	E°: 0.038–0.274 V pH > 8–11, 25 °C
Thiourea	S = C(NH_2_)_2_	Fe^3+^	[Au{S = C(NH_2_)_2_}_2_]^+^	E°: 0.38 V pH 1–2, 25 °C
Halide	Cl^−^	Cl_2_	[AuCl_4_]^−^	E^0^: 1.00 V pH < 4, 25 °C ^a^
	Br^−^	Br_2_	[AuBr_4_]^−^	
	I^−^	I_2_/I_3_^−^	[AuI_2_]^−^	

^a^ Leaching conditions for chlorine-chloride lixiviant system.

**Table 2:**
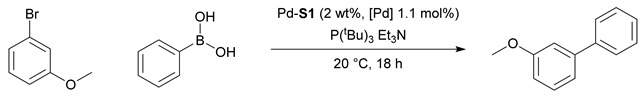
Catalytic activity of recovered micellar Pd catalyst, Reproduced with permission from [98].

Palladium Source	Yield (%)
Pd(OAc)_2_	85
MLCC recovered Pd	77

**Table 3:**
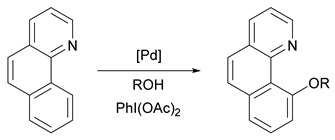
Catalytic activity of recovery product [Pd(Me_2_dazdt)_2_]I_6_ vs. conventional [Pd(OAc)_2_], Reproduced with permission from [106,108].

Pd(OAc)_2_ [108]				[Pd(Me_2_dazdt)_2_]I_6_ [106]			
Time (h)	Pd (mol%)	Temperature (°C)	Yield (%)	Time (h)	Pd (mol%)	Temperature (°C)	Yield (%)
18–24	1.2–5.1	100 °C	71–95	1–4	2	50 °C	79–100

## Data Availability

Not applicable.
